# Cost-effectiveness of controlling gestational diabetes mellitus: a systematic review

**DOI:** 10.1007/s10198-018-1006-y

**Published:** 2018-09-18

**Authors:** Najmiatul Fitria, Antoinette D. I. van Asselt, Maarten J. Postma

**Affiliations:** 1 Unit of Pharmaco-Therapy, -Epidemiology and -Economics (PTE2), Groningen Research Institute of Pharmacy, University of Groningen, A.Deusinglaan 1, 9713 AV Groningen, The Netherlands; 2 Unit of Pharmacology and Clinical Pharmacy, Faculty of Pharmacy, Universitas Andalas, Padang, West Sumatra Indonesia; 3 Department of Epidemiology, University Medical Center Groningen, University of Groningen, Groningen, The Netherlands; 4 Department of Health Sciences, University Medical Center Groningen, University of Groningen, Groningen, The Netherlands

**Keywords:** Hyperglycemia in pregnancy, Gestational diabetes mellitus, Cost-effectiveness

## Abstract

**Objective:**

Timely screening for hyperglycaemia in pregnancy using a simple glucose test enhances early detection and control of gestational diabetes mellitus (GDM). The aim of this study was to provide an overview of the evidence on the cost-effectiveness of identification and/or treatment of GDM.

**Methods:**

We conducted a systematic review using three electronic databases (PubMed, EMBASE, and Cochrane) of cost-effectiveness studies of GDM screening and treatment published during 2000–2017.

**Results:**

The initial search discovered 287 references (PubMed 86, EMBASE 195, Cochrane library 6) of which six full articles were included in the review. Two articles were model-based analysis and the remaining four were trial based. Two studies demonstrated favorable cost-effectiveness of intensified management of mild GDM. In the other included studies, neither screening nor treatment of GDM was shown to be cost effective, although results varied with the particular outcome measures used and the assumptions that where applied.

**Conclusion:**

Neither screening nor treating GDM seems to be convincingly cost-effective from the studies reviewed. However, all studies were done in high-income countries with obviously different health systems than low-/middle-income countries (LMIC) have. Since detection of GDM may be relatively poor in LMIC, screening might be more worthwhile in these countries. Comprehensive research is necessary in LMIC, including the potential outcomes of assessing its cost-effectiveness. Favorable cost-effectiveness could help in bridging the need for and access to increased diabetes screening in early pregnancy in these countries.

**Electronic supplementary material:**

The online version of this article (10.1007/s10198-018-1006-y) contains supplementary material, which is available to authorized users.

## Introduction

An increased blood glucose level (92–125 mg/dl) first detected at any time during pregnancy is classified as gestational diabetes mellitus (GDM) as part of hyperglycemia in pregnancy (HIP), which is any kind of increased blood glucose level during pregnancy, including live births in women with known diabetes [1]. The distinction between HIP and GDM has only recently (2013) been made by the World Health Organization (WHO) [2]. See supplementary Appendix 1 for an overview of the WHO classification.

The International Diabetes Federation (IDF) estimates that 21.4 million (16.8%) of women who gave live birth in 2013 had some form of HIP. There are some regional differences in the prevalence of HIP. The Southeast Asian Region had the highest crude incidence of the HIP at 23.1% of live births, followed closely by the Middle East and North African Region with 22.3% [3]. A staggering 91.6% of cases of the HIP were in low- and middle-income countries (LMIC). Estimates of GDM by region according to the diabetes atlas range from 10.4 to 25.0%, where North America-Caribbean is the lowest and Southeast Asia is the highest [1, 3]. Awareness of HIP as a risk factor and access to maternal care in LMIC are often limited.

GDM can significantly affect the health of both mother and child. A pregnant woman with diabetes can experience pre-eclampsia, infections, obstructed labor, and postpartum hemorrhage compared to women without diabetes [4–6]. These pregnant women with diabetes are also at risk of long-term complications associated with diabetes, such as retinopathy, nephropathy, and neuropathy [7, 8]. For the fetus, GDM is associated with stillbirth, preterm birth, macrosomia, growth retardation and congenital anomalies [9]. According to the American Diabetes Association, women with GDM should be screened for persistent diabetes at 6–12 weeks postpartum, and subsequently every 1–3 years [10]. An estimated 30–50% of women with a history of gestational diabetes develops it again in subsequent pregnancies within 5–10 years, and half of these women progress into type 2 DM [11]. Also, babies born from diabetic pregnancies are at increased risk of developing, for instance, juvenile obesity, metabolic disorders in adolescence and type 2 DM in adulthood [12]. The primary goal of managing all types of GDM is to create and maintain a normal blood glucose level for both the mother and fetus and also to prevent miscarriages and stillbirths [13–15]. GDM can be managed in many ways, for instance, using nutritional management, insulin treatment, or oral hypoglycemic agents [16–18]. According to the guidelines mentioned above, insulin is the first line of pharmacologic therapy.

Published data from IDF describe the majority of GDM screening is conducted in high-income countries (HIC) mainly in Europe and North America and Caribbean [3]. However, as the screening methodology used in HIC is more elaborate than commonly performed in LMIC, the evidence on GDM screening from HIC cannot be extrapolated to LMIC. Therefore, more data on screening for GDM in LMIC are needed to support the case for universal screening.

Treating the short- and long-term complications of GDM can be costly. Costs of treatment for perinatal complications in the United States may be up to US$9000 during the first year of life [19], and costs of treatment for T2DM can average up to US$3500 per year [20]. All strategies to reduce GDM require investments up front, and it should be determined whether these are worthwhile [21]. Cost-effectiveness analysis (CEA) compares the cost and effects of at least two strategies or interventions [22]. The outcome of a cost-effectiveness analysis is often an incremental cost-effectiveness ratio (ICER), which expresses the additional investments required to gain one additional unit of effect. Effects can be some measure of health such as the number of births at term, perinatal deaths prevented, or increased baby weight. In particular, quality-adjusted life years (QALYs) are often used [23, 24]. There have been many effectiveness trials but fewer cost-effectiveness studies in GDM. The objective of the present study is to provide, by means of a literature review, an overview of the existing evidence on the cost-effectiveness of identification and/or treatment of GDM.

## Methods

### Study design and search strategy

We conducted a literature review of cost-effectiveness studies related to gestational diabetes mellitus published between 2000 and 2017, taking into account reporting guidelines of preferred reporting items for systematic reviews and meta-analyses (PRISMA) diagram [25]. We decided to only include papers published from 2000 onwards as this is the first year after the diagnostic criteria for GDM were formally stated in 1999. We accessed three electronic databases (PubMed, EMBASE, and Cochrane) in August 2017. Supplementary Appendix 1 shows details of the search terms. We only included studies that were performed in pregnant women and that were written in English.

### Study selection and data extraction

The search results were downloaded into RefWorks Web-Based Bibliographic Management Software. From the initial search results, duplicates were removed, and title and abstract were screened. Articles that were not cost-effectiveness studies, not full papers (e.g., conference proceedings), or not on the topic of GDM were excluded. Alongside the data extraction, we converted cost estimates into a single currency (international $) and price year (2016), with the purpose of facilitating comparison of estimates collected from different studies. This conversion was performed using Organisation for Economic Co-operation and Development (OECD) Consumer Price Index and Purchasing Power Parities (PPPs) [26, 27].

### Quality of reporting

The Consolidated Health Economic Evaluation Reporting Standards (CHEERS) statement was used as a checklist to rate the quality of reporting in the included papers. The CHEERS statement of the International Society for Pharmacoeconomics and Outcomes Research (ISPOR) Health Economic Evaluation Publication Guidelines Good Reporting Practices Task Force is a guideline intended to improve reporting of economic evaluation [28, 29]. Within the CHEERS statement, a 24-item checklist is available to examine the quality of reporting of health economic studies.

### Risk of bias assessment

The recommended approach to assess risk of bias in reviews of cost-effectiveness studies is by means of the Consensus Health Economics Criteria (CHEC)-extended checklist [30, 31]. We chose to use a version that was adapted for specific use in diabetes mellitus type 2 (DMT2), as described in a study by Odnoletkova et al. [32]. This risk of bias approach was summarized using the Review Manager software.

## Results

### Systematic search strategy

**Fig. 1:**
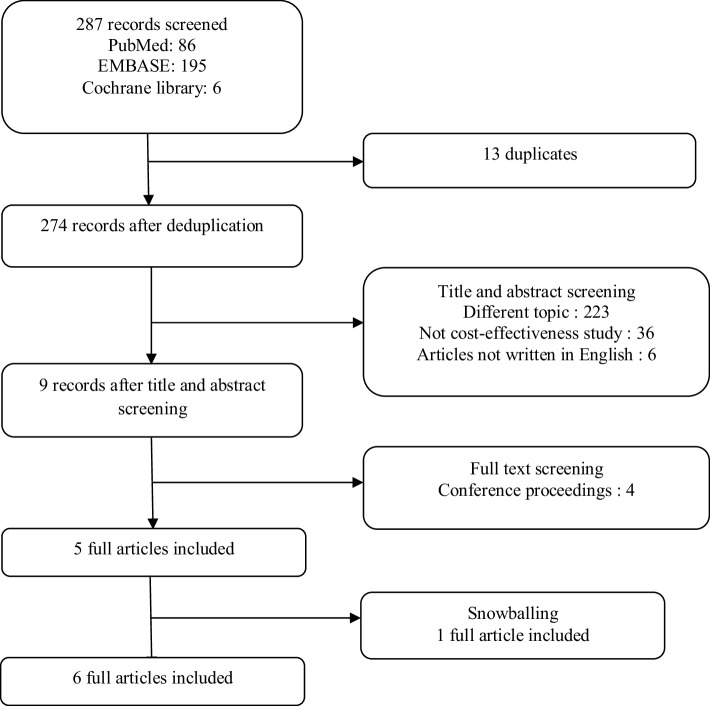
Flow of search strategy in systematic review

The database search discovered 287 references (PubMed 86, EMBASE 195, Cochrane library 6), of which 274 were left after deduplication (see Fig. 1 for a flow diagram). Screening of the title and the abstract found that 223 articles had a topic other than GDM, 36 articles were not cost-effectiveness studies, and 6 articles were not written in English. By this screening, nine articles met the inclusion criteria. Four of these articles were conference proceedings for which no full papers were available. Therefore, a final set of six publications was included in the study [33–38].

### Data extraction

An overview of the main study characteristics of the six included cost-effectiveness studies is provided in Table 1. Table 2 shows information on categories of included costs, currency and price year.

**Table 1:** Overview of main study characteristics of the included cost-effectiveness analyses on GDM management

Study	Study design						Analysis						
	Method	Perspective	Sample size	Countries	Treatment		Time horizon	Discount rate (%)		Sensitivity analysis	ICER/ NMB	ICER/ NMB (2016 I$)	Conclusion
					Intervention	Control		Cost	Effect				
Moss [33]	RCT (trial based)	Healthcare and patients	970	Australia	Dietary advice, monitoring blood glucose	Standard practice	9 months	5	5	Multi-variate, probabilistic sensitivity analysis	$27,503 per additional serious perinatal complication prevented, $60,506 per perinatal death prevented, $2988 per life/year saved	I$13,886.39 per additional serious perinatal complication prevented, I$30,549.77 per perinatal death prevented, I$1508.65 per life/year saved	The incremental cost per extra life-year gained is highly favorable at I$1,508,65
Ohno [34]	Model based	Healthcare	NA	United States of America	Nutritional counseling and diet therapy along with insulin (if required)	Usual prenatal care	Maternal plus neonatal lifetime	NM	3%	Univariate and probabilistic sensitivity analysis	$20,412 per QALY	I$23,745 per QALY	Treating mild GDM is cost effective below the cost-effectiveness threshold of I$116,326/QALY, as long as the cost to treat GDM was less than $4135
Oostdam [35]	RCT (trial based)	Societal	425	The Netherlands	Standard care + FitFor2	Standard care	9 months	NM	NM	Multi-variate, FCA and HCM	Fasting glucose: €46.97 per one point improvement in blood glucose Insulin sensitivity: €162.99 per unit improvement of IS HOMA QALY: inferior Birth weight: inferior	Fasting glucose: I$73.72 per one point improvement in blood glucose Insulin sensitivity: I$255.81 per unit improvement of IS HOMA QALY: inferior Birth weight: inferior	For fasting blood glucose and insulin sensitivity, the ICER of Fitfor2 was too high to be considered cost effective. For QALYs and birthweight, FitFor2 was inferior to standard care
Kolu [36]	Cluster-randomized trial (trial based)	Healthcare and societal	399	Finland	Insulin + lifestyle counseling	Standard care (insulin)	2 years	NM	NM	Multi-variate, probabilistic sensitivity analysis	€7 for increase in birth weight avoided (g)	I$9.27 for increase in birth weight avoided (g)	The intervention was effective in decreasing neonatal birth weight, but not cost effective for birth weight or quality of life
Kolu [37]	Cluster-randomized trial (trial based)	Healthcare and societal	173	Finland	Insulin + lifestyle counseling	Standard care (insulin)	7 years	NM	NM	Multi-variate, PSA	−€233 per day of absence from work prevented −€5386 per QALY	−I$258 per day of absence from work prevented −I$5974 per QALY	The intervention was not cost effective for QALY gained but may decrease the amount of sickness absence in women with risk of GDM
Farrar [38]	Model based, with four strategies compared	NHS and personal social services	NA	United Kingdom	No screening/testing or treatment		3 months	3.5	NM	PSA	NMB: −£1184	NMB: −I$1987	No screening/test or treatment is the least unfavorable among all scenarios at threshold I$33,573
					Screen only Screening followed by dietary and lifestyle advice for those who screen positive						NMB: −£1197	NMB: −I$2009	
					Universal diagnostic test Diagnostic test followed by dietary and lifestyle advice with pharmacological treatment as required						NMB −£1210	NMB: −I$2031	
					Screen and diagnostic test Screening followed by diagnostic test in those who screen positive, with dietary and lifestyle advice and pharmacological treatment as required						NMB: −£1197	NMB: −I$2009	

*ICER* incremental cost-effectiveness ratio, *NMB* net monetary benefit, *RCT* randomized control trial, *NM* not mentioned, *PSA* probabilistic sensitivity analysis, *QALY* quality-adjusted life year

**Table 2:** Cost categories which are taken into account in the included cost-effectiveness analysis study

Study	Categories of included costs	Currency, year
Moss [33]	Direct costs: antenatal clinic visits, specialist clinic visits, dietician visits, diabetes educator, blood glucose monitoring equipment, and insulin therapy Indirect costs: charges to the family: paid child care, travel, food substitution, mother time off paid work, and partner time off work	Australian dollars, 2002
Ohno [34]	Direct cost: pharmacotherapy, antenatal visits, ancillary diabetes-related visits, and antepartum fetal surveillance	US dollars, 2009
Oostdam [35]	Direct costs: general practitioner, medical specialist, hospitalization, occupational physician, mental health care, paramedical, dietician, midwife, obstetrician, delivery, and medications Indirect cost: productivity loss	Euro, 2009
Kolu [36]	Direct costs: laboratory test cost, health care visit cost, insulin/diabetes medication cost, delivery cost, hospital days cost, neonatal care cost, and costs of healthcare intervention: supplemental public health nurse’s contribution Indirect cost: Productivity loss	Euro, 2009
Kolu [37]	Direct costs: occupational health care, primary care doctor, special health care doctor, registered nurse, maternity clinic, family planning clinic, physiotherapist, and inpatient days in special health care Indirect cost: productivity loss	EUR, 2015
Farrar [38]	Direct costs: screening and diagnostic testing costs, adverse perinatal outcomes, treatment costs, and intensive lifestyle intervention costs	British Pounds, 2014

Four of the included studies were trial-based economic evaluations and two were model based. All trial-based studies in this review used intention-to-treat analysis. Clinical trials that use intention-to-treat analysis may be a reliable source for an economic evaluation, as they approximate real-world clinical practice better than per-protocol analyses [22]. Moss et al. compared dietary advice, blood glucose monitoring and insulin therapy as needed to routine pregnancy care in a population diagnosed with mild GDM [33]. Kolu et al. investigated the effect of lifestyle counseling compared to standard care among women at risk for GDM within 7 years of follow-up [36]. This study continued until 7 years of follow-up with half of the participants still included and the children who were born during the initial study [37]. Oostdam et al. also compared lifestyle counseling and scheduled exercise (FitFor2) in pregnant women at increased risk for GDM. Women in the control group were not presented the FitFor2 program and received care as usual [35].

All trial-based studies included reported an ICER for various outcome measures, e.g., birth at term, perinatal complications prevented, reduced birth weight in offspring, and QALYs. Moss et al. reported the ICER to be I$13,886 per-severe perinatal complication prevented and I$30,549 per perinatal death prevented. Even though fewer babies experienced perinatal complications and death, more women were induced into labor. Moss et al. also presented a long-term analysis based on simple extrapolation of the perinatal deaths prevented into life years gained. The incremental cost per life year gained was I$1508.65 which was considered to be highly cost effective. Kolu et al. present an ICER of I$9.27 for each additional gram of birth weight avoided. This intervention was effective in reducing birth weight, but also more expensive compared to usual care. After the 7-year follow-up, 70% of total costs in the population were due to absence from work. The intervention was not cost effective in terms of QALYs gained but still cost effective for absence from work with an ICER of −I$258 per day of absence from work prevented, indicating the dominance of the intervention as both costs were saved and absence from work was reduced.

In the study by Oostdam et al., the total cost in the intervention group was higher than for standard care because of prolonged hospitalization and a higher rate of preterm births in this group. This also caused a slight decrease in QALYs in the intervention group, implying the intervention was inferior, i.e., more costly and less effective, compared to standard care. Oostdam et al. also present an analysis on birth weight, which led to comparable results in the sense that most simulated cost-effectiveness pairs were in the northwest quadrant of the cost-effectiveness (CE) plane, so Fitfor2 was also considered inferior when it concerned reducing birth weight.

The study by Farrar et al. used a meta-analysis and modeling approach for the economic evaluation. They compared four strategies for testing and treating for hyperglycemia in healthy pregnancies. Their main results indicated that for the base case as well as for all scenarios analyses, the most cost-effective strategy at a £20,000 (I$33,573) threshold was ‘no screening/testing or treatment’. It is only with the inclusion of maternal longer term health outcomes and at cost-effectiveness thresholds of £24,000 (I$40,288) per QALY that net health benefits were improved by intervening. Ohno et al. also reported on a model-based study, comparing nutritional counseling, diet therapy plus insulin if required with usual prenatal care in women diagnosed with mild GDM [34]. The outcome for the economic evaluation was the sum of maternal and neonatal QALYs. Costs were also calculated from both the maternal and neonatal perspective, though only short-term events, i.e., related to pregnancy and delivery, were taken into account. Results indicated that treating GDM would be more expensive and more effective with an ICER of $20,412 per additional QALY, which was considered to be well below the threshold.

### Quality of reporting assessment

For each study, report on all 24 items in the CHEERS checklist is provided in the supplementary Appendix. Most of the studies reported quite comprehensively in the sense that they provide information on almost all items on the checklist. Moss et al. performed a trial-based economic evaluation and reported to have used bootstrapping to confirm their analysis. There is no report of the bootstrapping results, though, while an incremental cost-effectiveness plane or cost-effectiveness acceptability curve would have been informative as to the uncertainty surrounding outcomes.

### Risk of bias

**Fig. 2:**
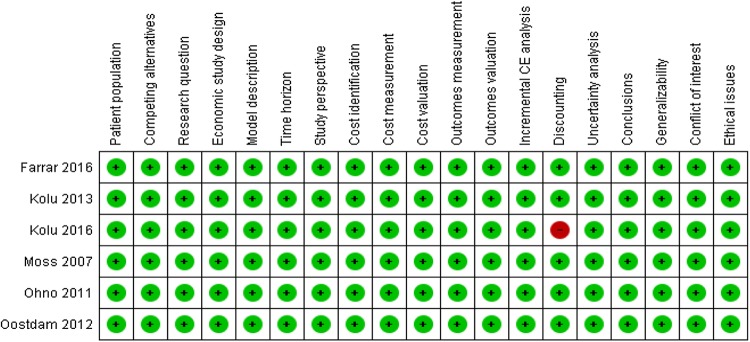
Risk of bias for each item of the modified CHEC-extended checklist

Figure 2 shows the summary information for risk of bias per study. It should be noted that for studies that were trial based, providing a model description was not applicable, so the absence of a description does not cause any bias. Also, when using a time frame for analyses of less than 1 year, discounting is not needed.

For trial-based economic analyses in gestational-based analyses, a follow-up from the early pregnancy until delivery that took less than 1 year would not be a problem in terms of discounting. Although Kolu et al. performed a long-term follow-up, they did not discount costs nor health effects. When they would have discounted future costs and health, the ICERs might have been impacted, although it is difficult to say in which direction.

The treatment estimates from Farrar were sourced from pooled RCT data of studies performed in HIC and, therefore, could likely validly be generalized to the UK obstetric population with GDM. In general, there were no serious structural sources or concerns for bias.

## Discussion

The inclusion criteria that we stated at the beginning of this study resulted in six articles included. The studies included in this review were exclusively located in high-resource countries. This is probably due to the fact that screening for GDM is common in these countries, as opposed to LMICs where screening programs are in the start-up phase, at best, and, therefore, economic evaluations are not yet in question.

In the studies included, several terms were used to describe standard care; standard practice, routine care and standard care itself. Their content could be different according to local guidelines in each hospital or study site. Differences in how standard care was defined and provided hamper comparison between the cost-effectiveness results of the included studies. The primary outcome of all studies was well defined.

In the countries and settings for which the economic evaluations were performed, maternity services and guidelines on screening and treatment of GDM were already well established. Pregnant women who were considered to be at a certain risk for GDM would have an HbA1c screening at 24–28 weeks of gestation [39]. A study by Jiwani in 2011 showed that more than 80% of countries that do not provide any GDM-related maternity care was LMIC [39]. They conclude that many of these countries have limited healthcare services capacity and do not yet have standardized practices for GDM screening and management.

Taking the evidence from all six papers together, it seems that treatment of GDM in itself may be effective, but screening the whole population for GDM and subsequently treat is not likely to be cost effective. According to Farrar et al. this is caused by the fact that the health benefits gained by treatment do not outweigh the investments needed to screen the whole population of pregnant women [38]. The unfavorable cost to benefit ratio may be a consequence of the fact that most GDM cases would, at a certain point, already be detected with care as usual and active screening does not significantly add to that. In this case then ‘no screening/testing or treatment’ is the cost-effective option at the considered range of cost-effectiveness thresholds [38]. Based on the small number of studies and sample sizes, the impact of screening women for GDM on health outcomes is inconclusive. The most commonly observed risk factors are age ≥ 30 years and family history of type 2 diabetes mellitus [38, 40]. However, in LMIC, the situation may be different. In LMIC the regimen of pregnancy checkups is less strict and occasional or regular detection of GDM may, therefore, be an exception. In this kind of situation, the added value of protocolized screening, as advocated by health authorities, would be higher.

One more reason for the somewhat disappointing cost-effectiveness of interventions directed toward GDM management might be that in all trial-based studies in this review, low compliance and high drop-out was a problem. As Oostdam et al. put it, ‘many women stopped exercising during the period of their pregnancy because of physical (pregnancy-related) limitations’ [35]. As it seems that the low compliance is intrinsic to the intervention and the pregnant population, it is unlikely that real-world cost-effectiveness would be better than reported from these trials.

Drawing conclusions from the included studies was difficult because of a number of reasons. First, the cost-effectiveness results were not always reported clearly and comprehensively. For instance, in the absence of an incremental cost-effectiveness plane, one has to very carefully check the results to see whether a negative ICER is the result of negative effects and positive costs, or the other way around, and when the outcome measure is expressed as ‘the less the better’ this complicates things even more. Furthermore, not all of the articles reviewed presented QALYs. Notably, the cost-effectiveness of screening or treatment is ideally reported in the way Ohno et al. have done [34], i.e., in terms of cost per QALY over the whole lifetime of both mother and child. The wide variety of outcome measures used in the included studies, even though perfectly relevant from a clinical point of view, adds to the inconclusiveness.

### Strengths and limitations

This is the first review to provide integrated evidence on cost-effectiveness in gestational diabetes. Next to summarizing results according to guidelines for systematic reviews of economic evaluation from van Mastrigt [30], we explicitly reported the risk of bias for all included studies. Combining trial- and model-based studies together in one table provides one integrated presentation, comparison and interpretation of the cost-effectiveness results. A definite limitation of this review is that some of the interventions investigated in the included studies were not yet proven to be clinically effective. Therefore, this review should not be used to conclude on the clinical effectiveness of therapeutic interventions, but rather be used to illustrate the potential favorable cost-effectiveness of interventions in gestational diabetes.

### Future research

While most countries can afford the investments needed, the poorest nations will need assistance to reach the targets. Even though WHO already provided the new screening approach, a standard estimation is still needed, as well as making cost-effectiveness analysis more generalizable to the LMIC. Since the sustainable development goals put attention on universal health coverage of reproductive, maternal, new-born and child health including service capacity and access, future research on this topic is warranted.

## Conclusion

From the included studies, GDM treatment could be considered cost effective under certain circumstances, but universal screening for GDM does not seem worthwhile. All studies in this review were done in high-income countries. Since regular detection of GDM is potentially poor in LMIC, the findings of this systematic review do not apply to an LMIC setting, and screening might be worthwhile in these countries. The decision on the best strategy for screening, diagnosis, and management should be made based on cost, availability, and accessibility of the local existing health facilities. Further research is warranted to assess applicability and cost-effectiveness concerning GDM especially in resource-limited countries of the world.

## Electronic supplementary material

Below is the link to the electronic supplementary material.

Supplementary material 1 (DOCX 14 KB)

Supplementary material 2 (DOCX 24 KB)

Supplementary material 3 (DOCX 16 KB)
