# Supplementary material for: Cost-effectiveness of controlling gestational diabetes mellitus: a systematic review
Source: Eur J Health Econ. 2018 Sep 18;20(3):407–17. doi: 10.1007/s10198-018-1006-y (PMC6438940; doi:10.1007/s10198-018-1006-y)
Supplement: Supplementary file 1 — Supplementary material 1 (DOCX 14 KB) [file 10198_2018_1006_MOESM1_ESM.docx]

| Pubmed | | EMBASE | | Cochrane Review | |
| --- | --- | --- | --- | --- | --- |
| #1 | "Diabetes, Gestational"[Mesh] OR gestational hyperglycaemia[tw] OR pregnancy diabetes[tw] OR pregnan* AND diabetes[tw] OR diabetes in pregnancy[tw] OR diabetes in pregnan*[tw] OR maternal diabetes[tw] OR maternal obesity[tw] OR GDM[tw] AND hyperglycaemia in pregnancy[tw] OR diabetes in pregnancy[tw] OR DIP[tw] | #1 | ‘gestational diabetes mellitus’/exp OR ‘gestational diabetes mellitus’ | #1 | [Diabetes, Gestational] explode all trees |
| #2 | ("Hypoglycemic Agents"[Mesh]) OR sulphonilurea[tw] OR sulfonylurea[tw] OR glyburide[tw] OR glyburid*[tw] OR glibenclamide[tw] OR glibenclamid*[tw] OR biguanide[tw] OR biguanid*[tw] OR metformine[tw] OR metformin*[tw] OR "Insulin"[Mesh] | #2 | ínsulin’ OR ‘hypoglycemic agent’ OR ‘antidiabetes drug’ OR ‘sulfonylurea’ OR ‘glyburide’ OR ‘glibenclamide’ OR ‘sulphonylurea derivative’ OR ‘biguanide’ OR ‘metformin’ OR ‘hypoglycaemic agent’ OR ‘hypoglycaemic drug’ OR ‘treatment’ | #2 | [Insulins] or [Hypoglycemic Agents] or [Metformin] or [Glyburide] or [Sulfonylurea Compounds] or [Biguanides] or [Treatment] |
| #3 | "Costs and Cost Analysis"[Mesh] OR "Cost-Benefit Analysis"[Mesh] OR "economics" [Subheading] OR cost[tw]OR costs[tw] OR cost of illness[tw] OR cost-utility[tw] OR cost-effectiveness OR economic eval*[tw] | #3 | ‘cost analysis’ OR ‘cost effectiveness analysis’ OR ‘cost benefit analysis’ OR ‘cost utility analysis’ | #3 | [Costs and Cost Analysis] explode all trees or [Cost-Effective Analysis] |
| #4 | ((("Diabetes, Gestational"[Mesh] OR gestational hyperglycaemia[tw] OR pregnancy diabetes[tw] OR pregnan* AND diabetes[tw] OR diabetes in pregnancy[tw] OR diabetes in pregnan*[tw] OR maternal diabetes[tw] OR maternal obesity[tw] OR GDM[tw] AND hyperglycaemia in pregnancy[tw] OR diabetes in pregnancy[tw] OR DIP[tw])) AND (("Hypoglycemic Agents"[Mesh]) OR sulphonilurea[tw] OR sulfonylurea[tw] OR glyburide[tw] OR glyburid*[tw] OR glibenclamide[tw] OR glibenclamid*[tw] OR biguanide[tw] OR biguanid*[tw] OR metformine[tw] OR metformin*[tw] OR "Insulin"[Mesh])) AND ("Costs and Cost Analysis"[Mesh] OR "Cost-Benefit Analysis"[Mesh] OR "economics" [Subheading] OR cost[tw]OR costs[tw] OR cost of illness[tw] OR cost-utility[tw] OR cost-effectiveness OR economic eval*[tw]) | #4 | #1 AND #2 AND #3 | #4 | #1 and #2 and3 |
|  |  |  |  |  |  |
|  | Result: 86 |  | Result: 195 |  | Result: 6 |

Appendix 1**.** Search strategy
