# Supplementary material for: Cost-effectiveness of controlling gestational diabetes mellitus: a systematic review
Source: Eur J Health Econ. 2018 Sep 18;20(3):407–17. doi: 10.1007/s10198-018-1006-y (PMC6438940; doi:10.1007/s10198-018-1006-y)
Supplement: Supplementary file 2 — Supplementary material 2 (DOCX 24 KB) [file 10198_2018_1006_MOESM2_ESM.docx]

Appendix 2**.** Hyperglycemia In Pregnancy classification (WHO,2013)
