# Supplementary material for: Cost-effectiveness of controlling gestational diabetes mellitus: a systematic review
Source: Eur J Health Econ. 2018 Sep 18;20(3):407–17. doi: 10.1007/s10198-018-1006-y (PMC6438940; doi:10.1007/s10198-018-1006-y)
Supplement: Supplementary file 3 — Supplementary material 3 (DOCX 16 KB) [file 10198_2018_1006_MOESM3_ESM.docx]

Appendix 3**.** Quality of reporting assessment CHEERS

| **Section/Item** | Moss (33) | Ohno (34) | Oostdam (35) | Kolu (36) | Kolu (37) | Farrar (38) | **Recommendation** |
| --- | --- | --- | --- | --- | --- | --- | --- |
| **Title and abstract** |  |  |  |  |  |  |  |
| Title | Y | Y | Y | Y | Y | Y | Title describes type of cost-effectiveness analysis |
| Abstract | Y | Y | Y | Y | Y | N | The abstract describes cost-effectiveness evaluation and intervention compared. |
| **Introduction** |  |  |  |  |  |  |  |
| Background and objectives | Y | Y | Y | Y | Y | Y | Provide research questions, the rationale of study, economic evaluation and research strategy (control and intervention). |
| **Methods** |  |  |  |  |  |  |  |
| Target population and subgroup | Y | Y | Y | Y | Y | Y | Base-case population /and criteria-based subgroup should fully describe. |
| Setting and location | Y | Y | Y | Y | Y | Y | State study sites (single-hospital, local, multi-city, regional or national) |
| Study perspective | Y | Y | Y | Y | Y | Y | Provide a point of view related to the cost evaluated. |
| Comparators | Y | Y | Y | Y | Y | Y | Provide the comparison of every intervention given |
| Time horizon | Y | Y | Y | Y | Y | Y | State the time-horizon which has a timeframe of at least one year. |
| Discount rate | Y | Y | NA | NA | N | Y | State discount rate in selected year. When timeframe less than one year, discounting is not needed. |
| Choice of health outcomes | Y | Y | Y | Y | Y | Y | Related to data collection, clinical outcomes analysis. Primary outcome well defined. |
| Measurement of effectiveness | Y | NA | Y | Y | Y | NA | For study/trial-based estimates, pay attention to randomized controlled study or observational study. |
|  | NA | Y | NA | NA | NA | Y | For model-based estimates, pay attention to combination using economic model |
| Measurement and valuation of preference-based outcomes | Y | Y | Y | Y | Y | Y | Data analyze refer to data collection methods whether it is trial or model based. |
| Estimating resources and costs | Y | NA | Y | Y | Y | NA | Use study-based economic evaluation |
|  | NA | Y | NA | NA | NA | Y | Use model-based economic evaluation |
| Currency, price date, and conversion | Y | Y | Y | Y |  | Y | Describe approximating quantities of unit costs, price date, and currency exchange rate. |
| Choice of model | NA | Y | NA | NA | NA | Y | The basic parameters in the decision-analytic model used are justified for model-based economic evaluation. |
| Assumption | Y | Y | Y | Y | N | Y | Use methods for estimating quantities and adjustment for the timing of cost. |
| Analytical methods | Y | Y | Y | Y | Y | Y | Describe all analytic methods support evaluation. |
| **Result** |  |  |  |  |  |  |  |
| Study parameters | Y | Y | Y | Y | Y | Y | Contains clinical outcome and value parameter |
| Incremental cost and outcomes | Y | Y | Y | Y | Y | Y | ICER is calculated when two strategies compared. |
| Characterizing uncertainty | Y | Y | Y | Y | N | Y | Depend on study-based economic evaluation or model-based economic evaluation. |
| Model-based economic evaluation | NA | Y | NA | NA | NA | Y | Use model-based in economic analysis. |
| Characterizing heterogeneity | N | Y | Y | Y | Y | Y | Describe pattern between subgroup analysis |
| **Discussion** |  |  |  |  |  |  |  |
| Study findings, limitations, generalizability, and current knowledge | Y | Y | Y | Y | Y | Y | Summarize research finding and restriction.  Conclude precisely the implication of information from the study. |
| **Other** |  |  |  |  |  |  |  |
| Source of funding | Y | Y | Y | Y | Y | Y | Describe how study was funded |
| Conflict of interest | Y | Y | Y | Y | Y | Y | Describe any potential conflict of interest |

Y-Yes, N-No, NA-Not Applicable.
